# Purple urine bag syndrome: case report

**DOI:** 10.31744/einstein_journal/2020RC5063

**Published:** 2019-09-16

**Authors:** Felipe Poubel Timm do Carmo, Alexandre Oliosi Caliman

**Affiliations:** 1 Santa Casa de Misericórdia de Vitória, Vitória, ES, Brazil.

**Keywords:** Urinary catheterization, Cystotomy, Urinary reservoirs, continent, Bacterial infections

## Abstract

A 65-year-old male with a history of urinary tract trauma requiring cystotomy and chronic bladder catheterization, presenting with chronic and uninvestigated changes in the color of the urine bag system, with no urine color change, and positive urine culture for *Proteus mirabilis* . These characteristics refer to the purple urine bag syndrome, a not weel-known condition, with a benign course in most cases, and associated with urinary tract infection in patients with chronic bladder catheterization. Although it is characterized by marked changes, it is underdiagnosed by healthcare professionals.

## INTRODUCTION

The purple urine bag syndrome (PUBS) is a fairly unknown condition.^( 1 )^ It is associated to urinary tract infection by bacteria that metabolize tryptophan into indigo and indirubin.^( 1 , 2 )^ These pigments precipitate inside the urine bag system, giving it a purple hue.^( 3 )^The known risk factors include older patients, female, patients in long-term care institutions, constipation and alkaline urine.^( 1 - 3 )^ Recognition and treatment are simple and important so that patients and family members can be educated to avoid excess management.^( 4 )^ In this article we report the case of a patient with this condition.

## CASE REPORT

A 65-year-old male obese patient, independent for activities of daily living, smoker and alcohol drinker as from 11 years of age (four cigarettes a day and 3 liters of sugarcane spirit/ *cachaça* per month). Patient reports falling from a tree (10 meter-high) 7 years ago, which resulted in pelvic trauma and hip fracture, and having an indwelling bladder catheter placed for an undetermined duration at the time. After removing the catheter, the patient had difficulty urinating; an urethrocystogram was performed in December 2012, showing an irregular urethra, with diffuse narrowing of the penile urethra. In October 2013, the patient evolved with urinary retention, hematuria and pyuria, in addition to testicular and anal injury, progressing to Fournier`s gangrene, requiring a suprapubic cystostomy. Since then the patient has been monitored in an outpatient setting, coming in to change the indwelling bladder catheter and the urinary bag every 30 days. In 2016, approximately 10 days after changes, the bag system started to show a bluish hue and, after 30 days, it turned purple, however, with no urine color change. During this interval, the patient was informed by healthcare professionals that the color changes were due to the ink used in the material of which the urine bag was made.

**Figure 1:**
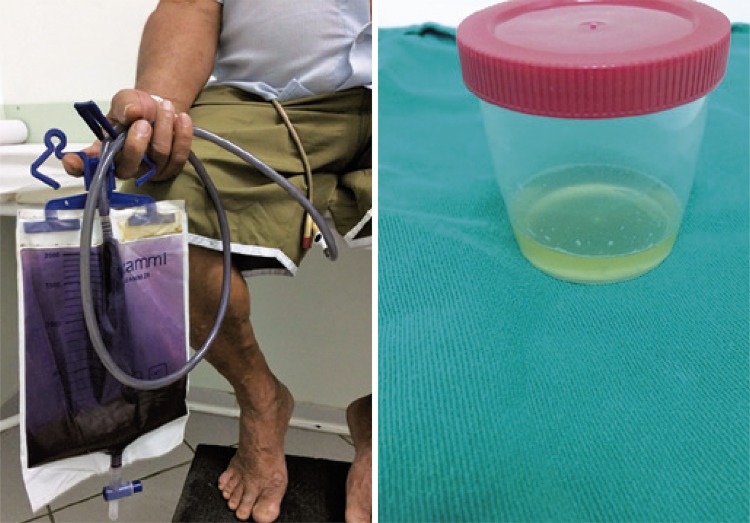
Purple urine bag

When evaluated for another change of the indwelling bladder catheter, the patient presented with a purple urine bag system ( Figure 1 ), yellow urine with a strong, foul smell, and pain in the hypogastrium. The patient`s diet was based on animal protein and fat, and he denied any comorbidities, use of medications, intestinal constipation, prior treatment for urinary tract infection, recent hospitalizations, and systemic symptoms. The patient had not taken proper care of the urine bag system, *e.g* . keeping it at the level of the shoulders, which led to backflow of contaminated urine into the bladder.

**Figure 2:**
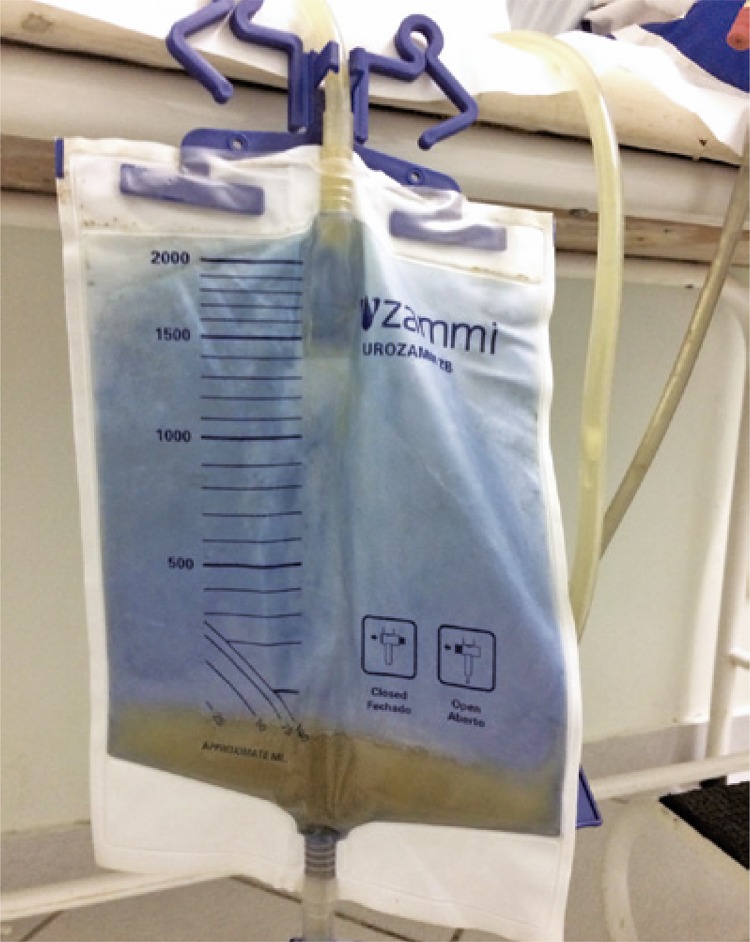
Purple urine bag system 15 days after catheter change

During the change, a sterile urine sample was collected and submitted for analysis and culture. He initiated treatment with trimethoprim-sulfamethoxazole, and a return visit was scheduled for 15 days later. Two weeks later, the urine bag had a bluish hue ( Figure 2 ) and laboratory tests showed abnormal elements and turbid sediments, bright yellow, density 1.005, pH 6.5, several pyocytes, red blood cells and negative nitrite. Also, the urine culture was positive for nitrofurantoin-resistant *Proteus mirabilis* .

**Figure 3:**
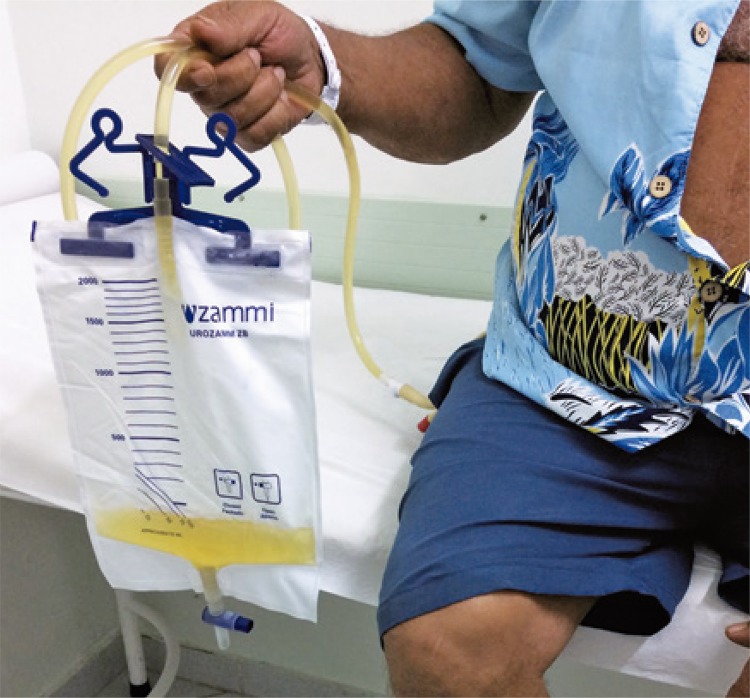
Purple urine bag system 30 days after treatment

Thirty days after collection, the urine still had a “bluish” hue, however less intense than before. Thus, treatment with ciprofloxacin was started, with 30-day control, and in the end, the patient had clear urine and the color of the urine bag system returned to normal ( Figure 3 ).

## DISCUSSION

The purple urine bag syndrome was first reported in 1978.^( 5 )^ It is a rare manifestation of urinary tract infection by bacteria that metabolize byproducts of tryptophan into red and blue pigments, of which the most common are *Providencia spp* ., *Escherichia coli* , *Proteus spp* ., *Pseudomonas spp* ., *Klebsiella pneumoniae* , *Morganella spp* . and *Enterococcus spp* . In the case reported, we detected the growth of *P. mirabils* .

Pigments are first produced in the gastrointestinal tract, where microbiome bacteria metabolize tryptophan into indole, which is transported in the bloodstream to the liver, where it is conjugated into indoxyl sulphate. It is excreted in urine, where bacterial sulfatases and phosphatases convert it into indoxyl which, in the presence of alkaline urine, is oxidated into indigo (blue) and indirubin (red).^( 2 , 4 , 6 )^ The literature shows that nearly 10% of long-term care patients with an indwelling bladder catheter are at risk of developing PUBS.^( 7 )^ The course of the syndrome is usually benign, although associated with higher morbidity and mortality, when compared to urinary tract infection alone.^( 2 , 7 )^ With this in mind, it is important to properly take the patient`s clinical history, since an erroneous diagnosis could lead to improper management of patients with PUBS. In the case reported, although the patient did not have many of the associated risk factors (constipation, older age, long-term care or alkaline urine), there was a clinical history clearly consistent with PUBS. However, since this condition is fairly unknown by healthcare professionals, the patient was erroneously informed that the purple color was from the ink of the bag system, which put the patient at risk due to lack of proper management.

## CONCLUSION

The purple urine bag syndrome is a fairly unknown condition, which leads to marked changes in the urine bag system due to bacterial metabolism in patients with indwelling bladder catheterization. Despite these changes, it is underdiagnosed by healthcare professionals unaware of the condition. Although the syndrome most often has a benign course, this is a risky situation since it is associated with urinary tract infection and higher morbidity and mortality among these patients.
