# Supplementary material for: Purple urine bag syndrome: case report
Source: Einstein (Sao Paulo). 2019 Sep 16;18:eRC5063. doi: 10.31744/einstein_journal/2020RC5063 (PMC6905158; doi:10.31744/einstein_journal/2020RC5063)
Supplement: Translation

# Translation: Síndrome do saco coletor de urina roxo: relato de caso

**Authors:** Felipe Poubel Timm do Carmo, Alexandre Oliosi Caliman

**Affiliations:** 1 Santa Casa de Misericórdia de Vitória, Vitória, ES, Brasil.

**Keywords:** Cateterismo urinário, Cistotomia, Coletores de urina, Infecções bacterianas

## Abstract

Homem de 65 anos com história de trauma do sistema urinário, sendo necessário cistotomia e sondagem vesical crônica, apresentando alterações crônicas e não investigadas da cor do sistema coletor de urina, sem alteração da cor da urina, e urocultura positiva para *Proteus mirabilis* . Tais características remetem à síndrome do saco coletor de urina roxo, uma entidade pouco conhecida, de curso benigno na maioria da vezes, associada à infecção urinária em paciente com cateterismo vesical de demora. Embora seja caracterizada por alterações marcantes, é subdiagnosticada pelos profissionais de saúde.

## INTRODUÇÃO

A síndrome do saco coletor de urina roxo (SSCUR) é uma entidade pouco conhecida.^( 1 )^Ela está associada à infecção urinária por bactérias, que metabolizam o triptofano em indigo e indirubina.^( 1 , 2 )^Esses corantes precipitam no sistema coletor de urina, deixando-o com uma coloração arroxeada.^( 3 )^Fatores de risco atribuídos são idosos, sexo feminino, pacientes institucionalizados, constipação e urina alcalina.^( 1 - 3 )^O reconhecimento e o tratamento são simples e importantes para que o paciente e os familiares sejam orientados, e o manejo excessivo seja evitado.^( 4 )^Neste trabalho, iremos relatar o caso de um paciente com essa condição.

## RELATO DE CASO

Homem de 65 anos, obeso, independente para atividades da vida diária, tabagista e etilista desde os 11 anos de idade (quatro cigarros por dia e 3L de cachaça por mês). O paciente refere queda de árvore (10 metros) há 7 anos, que provocou trauma pélvico com fratura de bacia, permanecendo com sonda vesical de demora por tempo indeterminado na época. Após retirar a sonda, apresentou dificuldade para urinar, sendo submetido à uretrocistografia em dezembro de 2012, evidenciando uretra irregular e com estreitamento difuso da uretra peniana. Em outubro de 2013, houve evolução para retenção urinária, hematúria e piúria, além de lesão em testículo e ânus, que evoluíram para síndrome de Fournier, necessitando de realização de cistotomia suprapúbica. Desde então, o paciente realiza acompanhamento ambulatorial, com troca de sonda vesical de demora e saco coletor a cada 30 dias. Em 2016, notou-se que aproximadamente 10 dias após as trocas, o sistema coletor passava a mudar sua coloração para um tom azulado, que, ao final de 30 dias, tornava-se arroxeado, porém, sem alteração da coloração da urina. Nesse intervalo, era informado por profissionais de saúde que as mudanças de tom seriam causadas pela tinta do material do saco coletor de urina.

Durante a avaliação para troca de sonda vesical de demora, o paciente apresentava sistema coletor roxo ( Figura 1 ), urina amarela e com odor fétido, e dor em hipogastro. Além disso, tinha alimentação baseada em proteína animal e gordura, e negava comorbidades, uso de medicamentos, constipação intestinal, tratamento anterior para infecção do trato urinário, internações recentes e sintomas sistêmicos. O paciente não realizava cuidados adequados com o sistema coletor de urina, por exemplo, acomodando-o nos ombros, o que permite o refluxo de urina contaminada para a bexiga.

**Figura 1:** Saco coletor de urina roxo

Durante a troca, foi colhida urina de forma estéril, que foi enviada para análise e cultura. A partir disso, foi iniciado o tratamento com sulfametoxazol e trimetoprima, com retorno programado para 15 dias depois. Após 2 semanas, o saco coletor apresentava tom azulado ( Figura 2 ) e os exames complementares apresentavam elementos anormais e sedimentos de aspecto turvo, amarelo claro, densidade de 1,005, pH de 6,5, numerosos piócitos, hemácias e nitrito negativo. Além disso, os exames apontaram urocultura positiva para *Proteus mirabilis* resistente à nitrofurantoína.

**Figura 2:** Saco coletor de urina roxo 15 dias após troca

Trinta dias após a coleta, a urina permaneceu com tom azulado, porém em menor intensidade que antes. Assim, foi iniciado o tratamento com ciprofloxacino, com controle de 30 dias, apresentando, ao final, urina clara e saco coletor sem alteração na coloração ( Figura 3 ).

**Figura 3:** Saco coletor de urina 30 dias após tratamento

## DISCUSSÃO

O primeiro relato médico da SSCUR foi em 1978.^( 5 )^Trata-se de uma manifestação rara de infecção do trato urinário por bactérias que metabolizam produtos do triptofano em pigmentos vermelhos e azuis, sendo as mais comuns, *Providencia spp* ., *Escherichia coli* , *Proteus spp* ., *Pseudomonas spp* ., *Klebsiella pneumoniae* , *Morganella spp* . e *Enterococcus spp* . No caso relatado, evidenciamos o crescimento da bactéria *P. mirabils* .

A produção de corantes começa no trato gastrintestinal, em que bactérias da flora metabolizam o triptofano em indol, que é transportado pela circulação até o fígado, onde é conjugado em indoxil sulfato. Este é excretado na urina, onde sulfatases e fosfatases bacterianas o convertem em indoxil, que, na presença de urina alcalina, é oxidado em indigo (azul) e indirubina (vermelho).^( 2 , 4 , 6 )^A literatura aponta que quase 10% dos pacientes institucionalizados e em uso crônico de cateter vesical de demora podem desenvolver a SSCUR.^( 7 )^O curso dessa síndrome é geralmente benigno, embora esteja associado à maior morbimortalidade, se comparada à infecção do trato urinário isolada.^( 2 , 7 )^Sabendo disso, é importante colher uma boa história clínica, visto que o diagnóstico equivocado pode levar à condução inadequada de pacientes com SSCUR. No caso relatado, apesar de não apresentar muitos fatores de risco associados (constipação, idade avançada, institucionalização ou urina alcalina), havia história clínica claramente compatível com SSCUR. Mas por se tratar de uma condição pouco conhecida pelos profissionais de saúde, o paciente era informado equivocadamente que a coloração estava associada à tinta do sistema coletor, o que ocasionou riscos ao paciente pela falta de tratamento adequado.

## CONCLUSÃO

A síndrome do saco coletor de urina roxo é uma entidade pouco conhecida, geradora de alterações marcantes no sistema coletor de urina, em decorrência de metabolização bacteriana em pacientes com cateterismo vesical de demora. Apesar dessas alterações, é subdiagnosticada por profissionais de saúde que desconhecem esta condição. Tal fato torna-se perigoso, pois, embora a síndrome tenha curso benigno na maioria das vezes, está associada a infecção urinária e maior morbimortalidade nesses pacientes.
